# Evaluation of normal appearing spinal cord by diffusion tensor imaging, fiber tracking, fractional anisotropy, and apparent diffusion coefficient measurement in 13 dogs

**DOI:** 10.1186/1751-0147-55-36

**Published:** 2013-04-24

**Authors:** Marc K Hobert, Veronika M Stein, Peter Dziallas, Davina C Ludwig, Andrea Tipold

**Affiliations:** 1 Department of Small Animal Medicine and Surgery, University of Veterinary Medicine Hannover, Buenteweg 9, 30559, Hannover, Germany

**Keywords:** Canine, Tractography, DTI, FA, ADC

## Abstract

**Background:**

Functional magnetic resonance (fMR) imaging offers plenty of new opportunities in the diagnosis of central nervous system diseases. Diffusion tensor imaging (DTI) is a technique sensitive to the random motion of water providing information about tissue architecture. We applied DTI to normal appearing spinal cords of 13 dogs of different breeds and body weights in a 3.0 T magnetic resonance (MR) scanner. The aim was to study fiber tracking (FT) patterns by tractography and the variations of the fractional anisotropy (FA) and the apparent diffusion coefficient (ADC) observed in the spinal cords of dogs with different sizes and at different locations (cervical and thoracolumbar). For that reason we added a DTI sequence to the standard clinical MR protocol. The values of FA and ADC were calculated by means of three regions of interest defined on the cervical or the thoracolumbar spinal cord (ROI 1, 2, and 3).

**Results:**

The shape of the spinal cord fiber tracts was well illustrated following tractography and the exiting nerve roots could be differentiated from the spinal cord fiber tracts. Routine MR scanning times were extended for 8 to 12 min, depending on the size of the field of view (FOV), the slice thickness, and the size of the interslice gaps. In small breed dogs (< 15 kg body weight) the fibers could be tracked over a length of approximately 10 vertebral bodies with scanning times of about 8 min, whereas in large breed dogs (> 25 kg body weight) the traceable fiber length was about 5 vertebral bodies which took 10 to 12 min scanning time. FA and ADC values showed mean values of 0.447 (FA), and 0.560 × 10^-3^ mm^2^/s (ADC), respectively without any differences detected with regard to different dog sizes and spinal cord 45 segments examined.

**Conclusion:**

FT is suitable for the graphical depiction of the canine spinal cord and the exiting nerve roots. The FA and ADC values offer an objective measure for evaluation of the spinal cord fiber integrity in dogs.

## Background

Currently, conventional magnetic resonance imaging (MRI) is the most widely used technique to examine the central nervous system (CNS). This technique enables the graphical depiction of structures such as the brain, the spinal cord, and the surrounding soft tissues [1-3]. Furthermore, pathological findings such as hemorrhages, edema, and physical injuries can be graphically depicted [4-6]. Pathological MRI changes of the spinal cord caused by compression appear as hyperintense lesions in T2 weighted images in conventional MRI [7]. These lesions might represent edema, hemorrhage but also necrosis or myelomalacia [7,8]. The histopathological correlate cannot be exactly determined *in vivo* by standard clinical MR protocol. Therefore, further imaging techniques are required to substantiate the prognosis in compressive spinal cord diseases.

Diffusion-weighted imaging (DWI) is a special technique of functional MR (fMR) imaging that has the capability to assess changes in random motion of water protons *in vivo*[9,10]. Diffusion tensor imaging (DTI) is an advanced technique of DWI that measures at least six diffusion directions and offers the possibility to track and graphically depict axonal fiber bundles by tractography [11-13]. DTI measures anisotropic water diffusion that occurs because of physiological borders such as axon bundles and myelin sheaths in the white matter of the brain and spinal cord [9,10,13].

DTI is of clinical importance presurgically in human medicine to plan function-preserving surgery by saving motor and speech reliable areas of the brain [14]. Moreover, technical advancement enables reconstruction of white matter tracts in 3D images not only of the brain but also of the spinal cord. For this purpose specialized fiber tracking (FT) algorithms are used to perform tractography, which depicts the spinal cord in three dimensions [11,12,14-20].

To prove the hypothesis that DTI with subsequent fiber tracking is suitable for visualization of the canine spinal cord and define reference values for the normal appearing canine spinal cord, DTI had to be applicable within a reasonable time frame added to a standard clinical MR protocol. DTI and subsequent fiber tracking were performed in the spinal cord of 13 dogs of different breeds to assess the integrity of spinal cord fibers. Additionally, fractional anisotropy (FA) and apparent diffusion coefficient (ADC) values were calculated for the three regions of interest (ROI 1, 2, and 3) on the ADC map at an Extended MR Workspace (Version 2.6.3.2 HF 3 2010, Philips Medical Systems). The FA value gives information about the directionality of the diffusion at each voxel and inferentially about the fiber integrity [9,21]. FA values represent so-called scalar values [9,22,23]. The ADC value describes the strength of the water diffusion and is composed of the values of all three diffusion directions (x-, y-, and z-axis). ADC values are described with the unit × 10^-3^mm^2^/s [9,21,24].

The aim was to study fiber tracking (FT) patterns by tractography and to evaluate the variations of the fractional anisotropy (FA) and the apparent diffusion coefficient (ADC) observed in normal appearing spinal cords of dogs with different sizes and at different regions (cervical and thoracolumbar) using a 3.0T MR scanner. These values will serve as reference values for comparison of fiber tract integrity in compressive spinal cord diseases to generate a potential additional prognostic tool.

## Methods

### Animals

Thirteen dogs of different breeds were included in the current study with the consent of the dog-owners. To fulfill the inclusion criteria, dogs had no clinical signs of an underlying disorder of the spinal cord and had normal appearing spinal cords as assessed by standard MR protocol. The 13 dogs underwent routine clinical MRI protocols under general anesthesia for diagnostic work-up of their suspected underlying disease (Table 1). Dogs were excluded if there was any suspicion of a neurological disorder affecting the spinal cord, history of diseases or previous surgery of the spinal cord. The study was conducted according to the ethical rules of the University of Veterinary Medicine Hannover and approved by the promotion Committee and the appointee for animal protection.

**Table 1:** Breed, body weight, age, diagnosis, and results of FA and ADC measurements of 13 dogs with normal appearing spinal cords

**Dog**	**Gender**	**Breed**	**Body weight (kg)**	**Age (months)**	**Diagnosis**	**FA (ROI 1)**	**FA (ROI 2)**	**FA (ROI 3)**	**ADC (ROI 1) (10**^**-3**^**mm**^**2**^**/s)**	**ADC (ROI 2) (10**^**-3**^**mm**^**2**^**/s)**	**ADC (ROI 3) (10**^**-3**^**mm**^**2**^**/s)**
**FT in the cervical spinal cord**											
1	f	Golden Retriever	30	93	osteosarcoma of the temporomandibular joint	0.448	nd	nd	0.413	nd	nd
2	m	American Staffordshire terrier	26	71	cerebellar abiotrophy	0.531	0.437	0.631	0.453	0.805	0.498
3	mc	English bulldog	16	27	head bobbing	0.338	0.381	0.464	nd	0.558	0.561
4	m	German wirehaired pointer	30	79	presumed neoplastic lesion in the right lobus occipitalis	0.291	0.337	0.316	nd	nd	nd
5	f	Mixed breed	14	66	idiopathic epilepsy	0.556	0.491	0.451	0.462	0.490	0.532
**FT in the thoracolumbar spinal cord**											
6	m	German wirehaired pointer	32	81	presumed neoplastic lesion in the right lobus occipitalis	0.512	0.409	0.527	0.857	0.782	0.811
7	fn	Griffon Bruxellois	6	75	Caudal occipital malformation syndrome	0.421	0.383	0.446	0.572	0.664	0.496
8	mc	Miniature poodle	7	21	lumbosacral stenosis	0.469	0.424	0.403	0.511	0.579	0.626
9	m	Miniature poodle	10	145	gastrointestinal foreign body	0.537	0.416	0.444	0.533	0.490	0.445
10	m	Airedale terrier	33	99	idiopathic epilepsy	0.550	0.502	0.493	0.526	0.541	0.521
11	f	Westhighland white terrier	7	11	idiopathic epilepsy	0.362	0.416	0.488	0.489	0.510	0.488
12	fn	Mixed breed	14	84	lymphoma in the spleen	0.594	0.46	0.387	0.217	nd	0.542
13	m	Malinois	27	46	orthopedic disease	0.409	0.40	0.401	0.654	0.62	0.624
**Mean**			**19.38**	**69.07**	**-**	**0.463**	**0.421**	**0.454**	**0.517**	**0.604**	**0.559**
**Median**				**75.00**	**-**	**0.469**	**0.416**	**0.449**	**0.511**	**0.569**	**0.532**
**Range**			**6-33**	**11-145**	**-**	**0.291-0.594**	**0.337-0.502**	**0.316-0.631**	**0.217-0.857**	**0.49-0.805**	**0.445-0.811**

gender: *m*; male, *f*; female, *mc*; male castrated, *fn*; female neutered, *FT*; fiber tracking, *FA*; fractional anisotropy, *ADC*; apparent diffusion coefficient, *nd*; not done (technical error).

### MRI

We used a 3.0T Philips Achieva MRI scanner (Philips Medical Systems) with one or a combination of two of the following different phased array coils: SENSE (sensitivity encoding)-spine-coil with 15 channels, SENSE-knee-coil with 8 channels, SENSE-neurovascular-coil TO with 8 channels, a combination in terms of dual-coil-imaging with the SENSE-neurovascular-coil and the SENSE-spine coil in large breed dogs.

The following parameters were used for acquisition of the T2-weighted sequences: TSE sequence, TR = 3000–6000 ms, TE = 80–120 ms, slice thickness 2.2-3.0 mm, with a 0.2-0.3 mm interslice gap, ACQ matrix size 256 × 204 in the protocol used for head scans with a reconstruction matrix of 512. In the protocol for thorax/abdomen scans an ACQ matrix of 448 × 333 with a reconstruction matrix of 1024 and 880, respectively was used. Twenty images were obtained so that the FOV varied from 180 to 273 mm according to the dog’s size. Voxel-size ranged from 0.7 × 0.88 × 2.2 to 0.91 × 2.2 × 2.2 and 0.91 × 3.0 × 3.0 mm, respectively.

### DTI/FT

A Reg DTI-high isoSENSE sequence was used for DTI (measurement method: EPI (echo planar imaging), DWI SE, repetition time: 12,000 ms; excitation time: 70 ms), with measurement of 33 diffusion directions (with a motion-probing gradient in 33 orientations). Sensitivity-Encoded (SENSE) reconstruction can be used to reduce the length of the EPI-train, thereby reducing susceptibility related distortions. Combining the EPI with the SENSE creates benefits in terms of reducing the disadvantages of EPI [25,26]. The FOV varied from 180 mm to 273 mm, according to the dog’s size. Number of b-values: 2, with a maximum b-factor of 800 mm^2^/s and an ascending b-factor order. Voxel-size: 2.0 × 2.0 × 2.0 mm. The ACQ matrix had a size of 122 × 111 (MxP) with a reconstruction matrix of 128. A total of 60–95 slices (thickness: 2.0-3.0 mm) with no interslice gap were obtained. Dynamic stabilization was used to improve image consistency across dynamics. The DTI sequence had a length of 8 to 12 min for each dog and was performed subsequently to the standard clinical MRI protocol. This protocol comprised T2-weighted (T2-w) sequences in sagittal and transversal planes, a transversal HEMO sequence and a fluid saturation sequence (Fluid Attenuated Inversion Recovery, FLAIR). Additionally, a dorsal plane was superimposed on the other planes when the head was scanned. In dogs with suspected intracranial diseases, the cervical spinal cord was scanned (dogs 1, 2, 3, 4, and 5). In eight dogs the thoracolumbar region (dogs 6, 7, 8, 9, 10, 11, 12, and 13) of the spinal cord was examined which is the region mostly affected by intervertebral disk disease in dogs [24]. T2-w images were used for orientation of the fiber tracking reconstruction to ensure the anatomical allocation of the reconstructed fibers. These T2-w images were imported into the DTI map. The subsequent tracking of the fibers, tractography, was performed on an Extended MR workspace (Version 2.6.3.2 HF 3 2010, Philips Medical Systems) with the special application tool “FiberTrak”.

**Figure 1:**
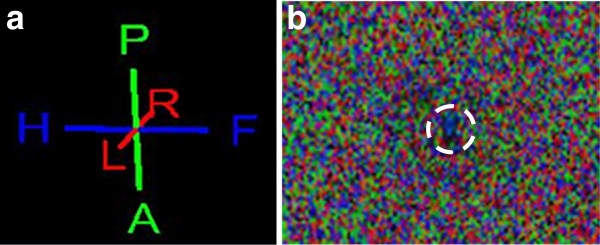
**a. Color-coding of the directional fiber tracking according to the course of water diffusion.** The data were color coded with blue indicating head-to-feet or craniocaudal direction, red denoting right-to-left (R-L) direction, and green indicating anterior-to-posterior (A-P) or dorsoventral orientation of water diffusion. **b**. **DTI raw data image with water proton diffusion coloring (as described above).** In the center (white circle) a relatively large blue colored area is visible caused by the craniocaudal course of water diffusion which is used to identify the spinal cord.

The DTI map depicts the spinal cord by use of the color-coded water diffusion direction and the anatomical orientation by implementing the related T2-w sequence into the DTI map (Figure 1a and b). The color-coded mapping assigns a specific color to the according direction of water. The water molecules within the axonal bundles of the spinal cord white matter prefer a craniocaudal or so-called head-feet diffusion direction. This direction is coded with blue color. Therefore, the spinal cord is depicted in blue (Figure 1b). The right-left direction is assigned to red color, and green is the color coding for the dorsoventral or posterior-anterior diffusion of the protons in water molecules (Figure 1a).

Tracking of the spinal cord fibers, so called tractography, allowed the graphical depiction of the spinal cord fibers and enables the determination of freely definable regions of interest (ROIs). Three ROIs, namely ROI 1, ROI 2, and ROI 3 (with 200 random seed points defined by the fully automated workstation) were set on the spinal cord itself (grey and white matter) with a distance of once to twice the length of a vertebral body in between the particular ROIs. By these selected anatomical regions the reproducibility of the measured values in the normal appearing spinal cord tissue of dogs should be proven. With special angle adjustments it was possible to depict not only the axonal bundles of the spinal cord itself but also the outgoing dorsal and ventral nerve roots graphically. The corresponding algorithm included minimal fractional anisotropy of 0.15, implying that the direction of diffusion anisotropy was followed until tracking was terminated when it reached a voxel with a FA of < 0.15. Furthermore, it included a maximal angle variation from 27.0° to 45° and a minimal length of the fibers of 10 mm. Directional fiber coloring and detailed illustration of the fibers was selected to achieve a more detailed graphical depiction of the axonal bundles. For each single ROI the corresponding FA- and ADC-values were calculated.

### Statistics

Testing for normal distribution of the data obtained was performed with the Kolmogorov-Smirnov and the Shapiro-Wilk normality tests. Differences between results of different ROIs were assessed by ANOVA with a Dunnett post-hoc test. An error probability of 0.05 (*P*) was used as significance level for all statistical tests.

## Results

Thirteen dogs of different breeds and ages with normal appearing spinal cords were imaged in a 3.0T MRI. FT studies allowed the graphical depiction of the axonal bundles of the cervical (*n* = 5) and thoracolumbar (*n* = 8) spinal cord by tractography in all 13 dogs. The shape of the bundles was string-like and well differentiable from the surrounding tissues. The spinal cord could only be scanned over a length of about 5 vertebral bodies in large breed dogs (> 25 kg body weight) and about 10 vertebral bodies in small breed dogs (< 15 kg body weight) due to scan time limitations and coil length. Accordingly, the FT scanning protocol took 8 to a maximum of 12 min in addition to the standard clinical MR protocol.

**Figure 2:**
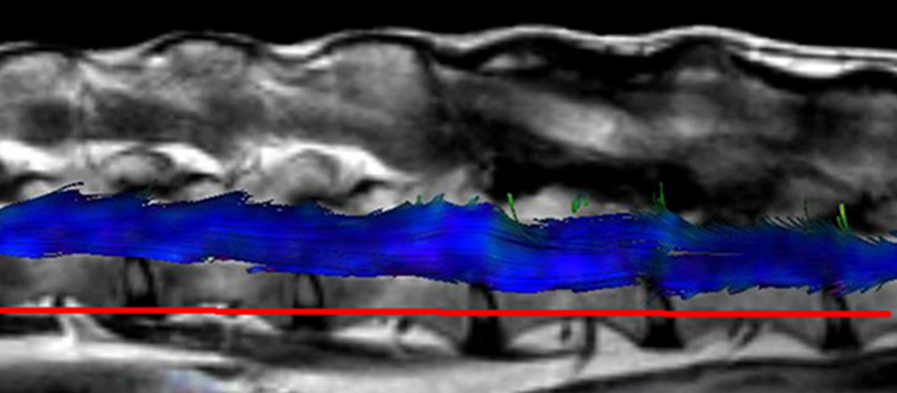
**Fiber tracking study of the lumbar spinal cord of a dog (no. 13) with the T2w sequence included (lateral view, left is cranial and right is caudal).** The axonal bundles of the spinal cord are string-like and appear mainly in blue color due to the mainly craniocaudal direction of water diffusion. Axonal bundles could be tracked over a length of approximately five vertebral bodies in this large breed dog.

The spinal cord DTI data generated were transferred to the Extended MR Workspace and displayed after 3D reconstruction. The main diffusion direction of water molecules within tissues defines the color coding in fiber tracking. Fibers are depicted in blue, red or green for craniocaudal, right-left or dorsoventral orientation, respectively (Figure 1a). As water molecules within the spinal cord prefer a craniocaudal diffusion direction along white matter fibers they are illustrated mainly in blue color (Figure 1b and Figure 2).

**Figure 3:**
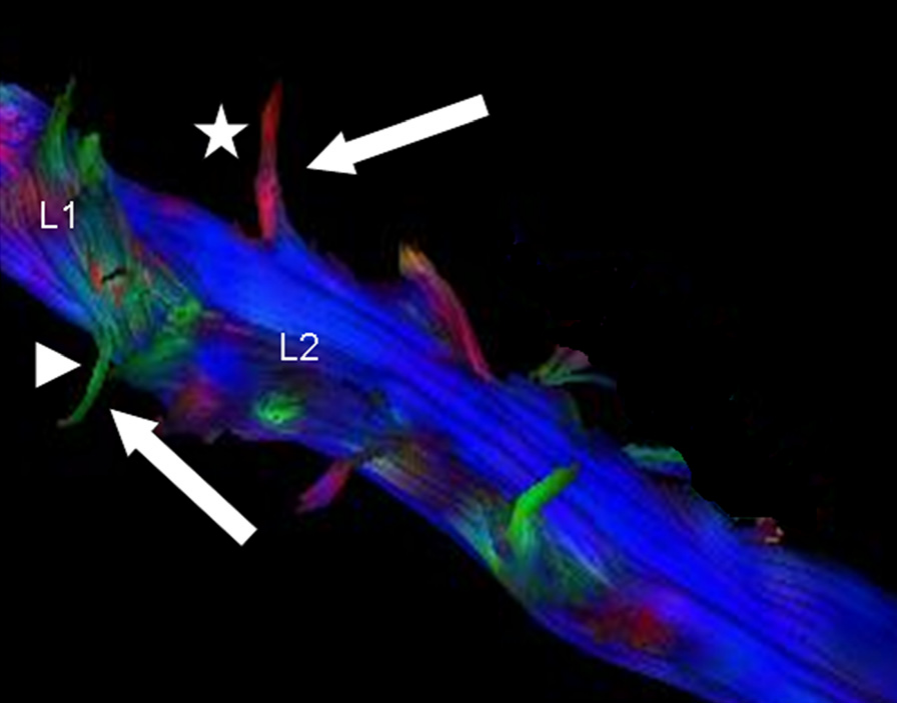
**Blue color-coded axonal bundles of the L1 to L4 spinal cord segment.** The exiting green and red fiber bundles (white arrows) of the first and second lumbar spinal segment (L1 and L2) represent the exiting dorsal (green, arrowhead) and ventral (red, star) nerve roots. Color-coding is according to the main direction of water diffusion. Maximum angle variation 45.0°. For a better visualization of the exiting fiber bundles the image of the spinal cord was rotated.

Interestingly, the exiting nerve roots were not depictable in all dogs with the standard adjustments (maximum angle variation of 27.0°). Hence, the maximum angle variation had to be modulated. Increasing the maximum angle from 27.0° to 45.0° allowed graphical depiction of both, the ventral and the dorsal nerve roots exiting the vertebral canal through the intervertebral foramen in all 13 dogs. The ventral nerve roots were colored green due to the mainly dorsoventral or anterior-posterior diffusion direction, whereas the dorsal nerve roots were coded in red because of their mainly right-left course (Figure 3).

**Figure 4:**
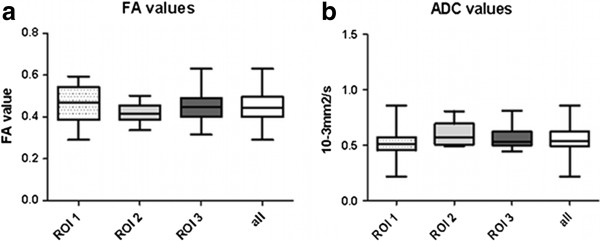
**Results of fractional anisotropy (FA) and apparent diffusion coefficient (ADC) measurements in spinal cords of 13 dogs. a**. FA values were assessed for three different regions of interest (ROI) with ROI 1 (dotted box), ROI 2 (pale grey box), and ROI 3 (dark grey box) representing the values of the FAs at the different ROIs. No statistically significant difference occurred between ROIs (*P* = 0.8045). The box on the right (white) comprises the FA values of all 13 dogs together. **b**. ADC values were evaluated for the three defined ROIs, ROI 1 (dotted box), ROI 2 (pale grey box), and ROI 3 (dark grey box). No statistically significant difference between ROIs could be detected (*P* = 0.9688). The boxes contain the middle 50% of the sample values, Tukey boxplots display minimum and maximum, lower and upper quartiles, and median, and contain 95% of the values. FA = fractional anisotropy, ADC = apparent diffusion coefficient, ROI = region of interest, all = values of all 13 dogs and cervical and thoracolumbar spinal cord comprised.

Fractional anisotropy (FA) and apparent diffusion coefficient (ADC) were assessed for 13 healthy dogs of different breed and at different spinal cord segments. The mean FA value in the normal appearing spinal cords was 0.447 with a range of 0.291 to 0.631 and a standard error of 0.0163 (Table 1). The median of the FA was 0.448 (Figure 4a). The median of the ADC value in normal appearing spinal cords was 0.532 × 10^-3^mm^2^/s and the mean ADC value was 0.560 × 10^-3^mm^2^/s with a range from 0.217 to 0.857 × 10^-3^mm^2^/s and a standard error of 0.0126 (Figure 4b, Table 1). No statistically significant difference could be demonstrated between different sites of the spinal cords measured by the three ROIs (*P* (FA) = 0.8045 and *P* (ADC) = 0.9688). Evaluation of FA and ADC did also not reveal a statistically significant difference between small and large breed dogs (*P* (FA) = 0.787 and *P* (ADC) = 0.143).

## Discussion

So far, DTI was applied presurgically in human medicine to plan function-preserving brain surgery by saving special areas of motor and speech function [14]. Nowadays the application of this technique is also possible for white matter tract reconstruction in 3D images for the spinal cord [11,12]. Specialized fiber tracking (FT) algorithms allow graphical depiction of the spinal cord in three dimensions [14,15].

This study shows that the technique of diffusion tensor imaging (DTI) with subsequent tractography is applicable to dogs of different breeds and size/body weight and allows the graphical depiction of canine spinal cord in a remarkably detailed explicitness at high resolution, respectively the water diffusion along the axonal bundles. The scans were performed with a 3.0 Tesla (T) MR scanner. In a previous study by Pease and Miller a 1.5 T scanner was used and beagles were used as controls [20]. The authors could show that the technique of fiber tracking is feasible in dogs. In the current study DTI was performed with a 3.0 T scanner, which offers more detailed precision. A short scanning time as offered by the 3.0 T scanner is necessary to introduce the technique into the daily clinical practice. Furthermore, reference values were established for a different scanner type and for dogs of different breeds and sizes. A special sequence, termed “*Fac Reko Reg DTI-high iso SENSE”,* with a duration of 8 to 12 min was added to the routine clinical protocol. The scanning time of this additional sequence did not excessively extend the overall scanning time and can therefore be added to the clinical standard protocol to serve as a prognostic factor. For analysis of the DTI data specialized software is required. This software was part of the corresponding MR work space. The field of view (FOV) for the DTI-scan was defined on the sagittal section plane. This was sufficient because the post-processing on the work station assembled a three dimensional (3D) reconstruction data set. Hence, the tractography can be rotated in all directions in this 3D-reconstruction.

The FA values of all 13 scanned dogs with normal appearing spinal cords showed a range of 0.291 to 0.631 independent of the measured spinal cord segment (ROI 1, 2, and 3). The results of the measurements were reproducible and the values did not differ significantly between the ROIs. Breed and body weight of the dogs had no influence on the distribution of measured values. The FA mean value determined in the current study (0.447) is lower compared to the mean value described by Pease and Miller [20]. However, in the study by Pease and Miller FA values were recorded in only one medium-sized dog breed and only in one spinal region, the cervical spinal cord. Values of the current study are comparable with data established for healthy cats ranging from 0.36 to 0.62. [27]. The ADC values of the current study showed a wider range than the FA values, namely from 0.217 to 0.857 × 10^-3^mm^2^/s. Pease and Miller detected comparably higher ADC values with a mean of 1.0253 × 10^-3^mm^2^/s for their control group consisting of eleven normal Beagle dogs scanned in the cervical region of the spinal cord [20]. Furthermore, the inclusion of surrounding cerebrospinal fluid and fat in the ROIs leads to bias by producing significantly lower FA values [28].

Differences might be explained by the scanner used. Therefore, reference values should be establisned for each scanner and software used for the measurements. Single ROI measurements were not evaluable in four dogs (see Table 1) because of a technical problem.

## Conclusions

DTI data not only offer the possibility to depict axonal bundles of the spinal cord and the exiting nerve roots in a graphically three-dimensional way but also to assess the axonal bundles by definite values, such as FA and ADC. These objective values provide further important information about the directionality of the diffusion (FA) and the strength of the diffusion itself (ADC) within the neuronal structures of the CNS. Objective values to characterize the spinal cord are comprehensible using fMRI.

These additional data can be used therefore for further studies to evaluate spinal cord injuries in dogs and may reveal abnormalities that are occult on conventional MRI scans.

## Abbreviations

ACQ matrix: Acquisition matrix; ADC: Apparent diffusion coefficient; A-P: Anterior-to-posterior; CNS: Central nervous system; DTI: Diffusion tensor imaging; DWI: Diffusion-weighted imaging; EPI: Echo planar imaging; FA: Fractional anisotropy; FLAIR: Fluid attenuated inversion recovery; fMR: Functional magnetic resonance; fMRI: Functional magnetic resonance imaging; FOV: Field of view; FT: Fiber tracking; nd: Not done; MR: Magnetic resonance; R-L: Right-to-left; ROI: Region of interest; SE: Spin echo; SENSE: Sensivity encoding; T: Tesla; T2-w: T2-weighted; TE: Echo delay time; TR: Repetion time; TSE: Turbo spin echo

## Competing interests

None of the authors of this paper has a financial a personal relationship with other people or organisations that could inappropriately influence or bias the content of the paper. None of the authors of this manuscript have any competing interest to declare.

## Authors’ contributions

MKH, PD, and DL performed the fiber tracking studies. MKH, VMS, and AT conceived the study, participated in its design and study coordination. MKH performed the statistical analysis, and drafted the manuscript. All authors read and approved the final manuscript.
